# Update on the Utility of Optical Coherence Tomography in the Analysis of the Optic Nerve Head in Highly Myopic Eyes with and without Glaucoma

**DOI:** 10.3390/jcm12072592

**Published:** 2023-03-29

**Authors:** Bachar Kudsieh, José Ignacio Fernández-Vigo, Ignacio Flores-Moreno, Jorge Ruiz-Medrano, Maria Garcia-Zamora, Muhsen Samaan, Jose Maria Ruiz-Moreno

**Affiliations:** 1 Department of Ophthalmology, University Hospital Puerta De Hierro Majadahonda, 28220 Madrid, Spain; 2 Centro Internacional de Oftalmologia Avanzada, 28010 Madrid, Spain; 3 Department of Ophthalmology, Hospital Clinico San Carlos, Institute of Health Research (IdISSC), 28040 Madrid, Spain; 4 Instituto de Microcirugia Ocular (IMO), 28035 Madrid, Spain; 5 Barraquer Eye Clinic UAE, Dubai P.O. Box 212619, United Arab Emirates

**Keywords:** optic nerve head, high myopia, myopic glaucoma, optical coherence tomography, peripapillary atrophy, optic disc tilt, intrachoroidal cavitation, retinal nerve fiber layer

## Abstract

Glaucoma diagnosis in highly myopic subjects by optic nerve head (ONH) imaging is challenging as it is difficult to distinguish structural defects related to glaucoma from myopia-related defects in these subjects. Optical coherence tomography (OCT) has evolved to become a routine examination at present, providing key information in the assessment of glaucoma based on the study of the ONH. However, the correct segmentation and interpretation of the ONH data employing OCT is still a challenge in highly myopic patients. High-resolution OCT images can help qualitatively and quantitatively describe the structural characteristics and anatomical changes in highly myopic subjects with and without glaucoma. The ONH and peripapillary area can be analyzed to measure the myopic atrophic-related zone, the existence of intrachoroidal cavitation, staphyloma, and ONH pits by OCT. Similarly, the lamina cribosa observed in the OCT images may reveal anatomical changes that justify visual defects. Several quantitative parameters of the ONH obtained from OCT images were proposed to predict the progression of visual defects in glaucoma subjects. Additionally, OCT images help identify factors that may negatively influence the measurement of the retinal nerve fiber layer (RNFL) and provide better analysis using new parameters, such as Bruch’s Membrane Opening-Minimum Rim Width, which serves as an alternative to RNFL measurements in highly myopic subjects due to its superior diagnostic ability.

## 1. Introduction

Population-based studies have shown that myopia is a major risk factor for open-angle glaucoma (OAG) [1,2]. Furthermore, the morbidity rate of glaucoma increases along with the degree of myopia [3]. Hence, the diagnosis of glaucoma in the early stages of the disease in highly myopic eyes is essential for the management and prevention of visual loss.

In highly myopic subjects, the image-based diagnosis of glaucoma is challenging as it is difficult to distinguish structural defects related to glaucoma from myopia-related defects [4]. Optical coherence tomography (OCT) is widely used to measure the optic nerve head (ONH) neuroretinal rim and the peripapillary retinal nerve fiber layer (RNFL), both being the most popular parameters to distinguish glaucoma by clinicians [5].

The anatomical changes caused by the progressive increase in the axial length (AXL) in myopic subjects lead to abnormal positions of the ONH, such as optic disc tilting and torsion [6]. These phenomena result in difficulties in applying the ISNT rule in highly myopic subjects to diagnose the presence of glaucoma [7]. Similarly, in high myopia, the trajectories of the peripapillary nerve fiber layer deviate, hindering the interpretation of OCT measurements [8,9]. Additionally, the visual field (VF) defects in myopic glaucoma subjects are confusing due to their co-occurrence with myopic chorioretinopathy VF defects, making the relation between structural and functional defects in myopic glaucoma weak [10]. The advancement of Swept Source (SS) and Enhanced Depth Imaging (EDI) OCT has enabled clinicians to obtain high-resolution images of the ocular structures, including the deepest layers, which is useful in highly myopic subjects [11]. Through these images, the papillary area can be qualitatively and quantitatively described and these findings can be associated with visual function.

In this review, we summarize and comment on the utility of OCT in identifying and measuring the structural variations and abnormalities of the ONH and peripapillary region in highly myopic subjects with and without glaucoma, including the quantitative and qualitative characteristics of the OCT measurements and the relation of these changes with VF.

In this review, a PubMed search was undertaken in January 2023 using the following search terms: “high myopia”, “optic nerve head”, “optical coherence tomography”, and “glaucoma” and combinations. The abstracts were screened and those relevant to this particular review were retrieved for more detailed analysis.

## 2. Peripapillary Atrophy

**Figure 1:**
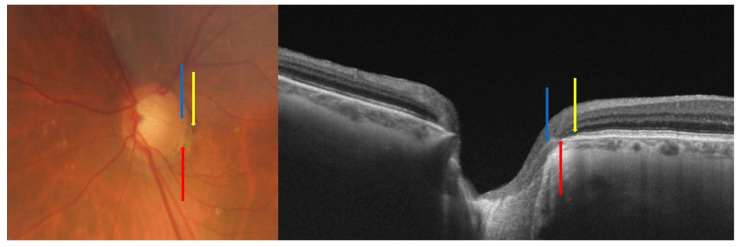
Fundus photography and optical coherence tomography of the peripapillary region of a myopic subject. The blue arrow represents the temporal optic disc margin, the red arrow represents the end of Bruch’s membrane (BM), and the yellow arrow represents the end of the retinal pigment epithelium (RPE). The distance from the temporal optic disc margin (blue arrow) to the edge of BM (red arrow) is defined as peripapillary atrophy (PPA) beta and the distance from the edge of BM (red arrow) to the end of RPE is defined as PPA alpha.

In the peripapillary area of highly myopic subjects, four zones of peripapillary atrophy (PPA) can be differentiated using radial scans of OCT images [12] (Table 1, Figure 1). The alpha PPA (α-PPA) consists of an irregular retinal pigment epithelium (RPE) with the presence of a Bruch’s membrane (BM). This area coincides with the clinically observed PPA by retinography. The beta PPA (β-PPA) is characterized by the absence of RPE and the presence of BM. A larger β-PPA area is associated with older age, longer AXL, larger disc area, greater disc ovality, and thinner choroidal thickness [13]. Its presence in glaucoma patients indicates a greater risk of progression and major VF loss [14]. The gamma PPA (γ-PPA) is characterized by the absence of both the RPE and BM, with only the presence of the peripapillary RNFL. This type of PPA is characteristic of myopia and is usually caused by a progressive elongation of the eye regardless of glaucoma. In highly myopic eyes with AXL greater than 26.5 mm, the optic disc area, lamina cribosa (LC) area, and BM opening area increase, leading to a circular γ-PPA and δ- PPA [15]. The delta PPA (δ-PPA) is characterized by the absence of microvessels larger than 50 microns. It is usually within the γ-PPA zone, predisposes to glaucomatous damage, and coincides with the dura mater insertion into the sclera [13,16,17]. The size of the PPA area is usually associated with the AXL, age, and choroidal thickness. Hu et al. described a PPA area of 0.35 mm^2^ in subjects with AXL below 24 mm, 0.65 mm^2^ when the AXL is between 24 and 27 mm, and 0.78 mm^2^ in high myopia (AXL greater than 27 mm^2^) [18]. Similarly, in a cross-sectional study including 821 young myopic patients, Chen et al. discovered that every 0.1 mm^2^ increase in the PPA area was associated with a 14.93 μm decrease in the macular choroidal thickness (MCT) and a 9.54 μm decrease in the peripapillary choroidal thickness [17]. On the contrary, the PPA area has a negligible or weak correlation with the RNFL thickness [17,18]. Hu et al. found that the PPA area does not usually correlate with the RNFL thickness [18]. In agreement with this, a mild correlation between the PPA and RFNL thickness (R = 0.417, *p* < 0.001) was described by Zhang et al. in a study of 112 patients with a mean AXL of 26.86  ±  0.94 mm [19].

## 3. Tilted Disc in High Myopia

**Figure 2:**
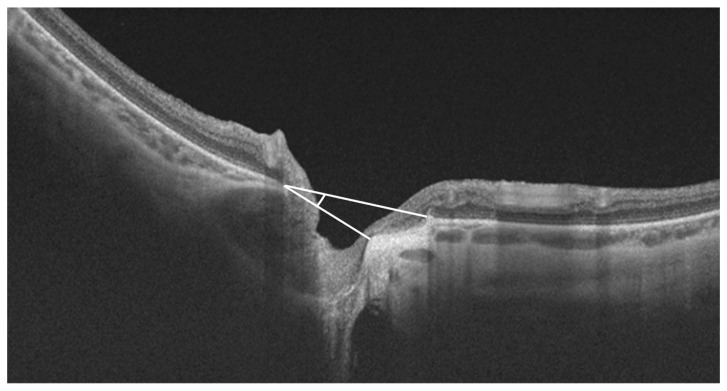
Optic nerve head tilt angle measurement in myopic subject. The tilt angle represents the angle is between the upper white line connecting the inner edges of Bruch’s membrane on each side of the optic nerve head on the cross-sectional optical coherence tomography (OCT) image, and the lower white line connecting the two points marking the clinical disc margin along the OCT cross-sectional scan.

The tilt of the ONH represents an anomalous insertion of the optic nerve in the eyeball in highly myopic subjects. It can be horizontal or vertical, and it has traditionally been evaluated in the images obtained with retinography with the ovality index [20,21]. Recently, the optic nerve tilt angle has been accurately measured in vertical and horizontal scans of OCT images [22]. Hosseini et al. defined the tilt angle as the angle between two lines: the first line connecting the inner edges of the BM on each side of the ONH on the cross-sectional OCT image, and a second line connecting the two points marking the clinical disc margin along the OCT cross-sectional scan (Figure 2). In that study, the median tilt angle was 3.5° (1.2–11.2) [22]. Similarly, Choi et al. found a median temporal and vertical disc tilt using a spectral domain (SD) OCT of 3.60° (1.61° to 6.40°) and 0.00° (−1.03° to 1.62°), respectively, in their study, which included 235 eyes of normal and glaucoma patients with a mean AXL of 24.5 mm. Moreover, they obtained excellent intra- and inter-observer reproducibility for the angle measurements by OCT (ICC = 0.882 and 0.801, respectively) [23]. Tilted optic discs are more frequent in myopic eyes. A study of young myopic subjects revealed that the frequency of tilted optic discs increased with the AXL, being 37.0%, 51.1%, 57.6%, and 70.7% with AXL  ≤  24 mm, 24 to 25 mm, 25 to 26 mm, and ≥26 mm, respectively [17]. Hosseini et al. demonstrated a positive correlation between the disc tilt angle and AXL (R = 0.399, *p* < 0.001) [22]. A greater tilt in myopic eyes with glaucoma was suggested by Park et al. who observed a significantly greater tilt angle in myopic glaucoma compared to control eyes with similar AXL, being 9.3 ± 6.3° vs. 6.2 ± 4.1°, respectively (*p* < 0.05) [24]. Similarly, Yoon J. Y. et al. found that young myopic glaucomatous eyes showed progressive optic disc tilting during three years of follow-up, increasing from 7.0 ± 3.4 to 8.3 ± 3.8° [25]. The authors attribute these findings in myopic eyes with glaucoma to either continuous AXL enlargement or/and glaucomatous structural change.

The correlation between disc tilt in myopic patients and VF defects has been described by several authors. Hosseini et al. demonstrated a negative correlation with VF defects (R = −0.356, *p* < 0.001). Shoeibi et al. showed arcuate scotoma and generalized depression on the VF in 30% and 30% of myopic eyes with a tilted disc, respectively, observing the lowest average deviation of the VF in the superotemporal quadrant (−4.54 ± 3.16 dB) [26]. In addition, Han et al. described a faster VF progression in myopic OAG with inferiorly tilted discs compared to non-myopic OAG (*p* = 0.002) [27].

Recent studies using OCT have demonstrated that the assessment of the ONH and RNFL are strongly influenced by the optic disc tilt. Refs. [28,29] Shin et al. showed that the disc area, cup volume, and average cup-to-disc-ratio (CDR) obtained by Cirrus HD-OCT were significantly smaller in myopic tilted eyes compared to the non-tilted myopic eyes (1.91 ± 0.81 mm^2^ vs. 1.63 ± 0.30 mm^2^, 0.53 ± 0.28 mm^2^ vs. 0.33 ± 0.19 mm^2^ and 0.76 ± 0.09 vs. 0.69 ± 0.11, respectively, all *p* < 0.05). Similarly, they obtained thicker temporal RNFL in the tilted disc group compared with the non-tilted group (62.1 ± 9.0 μm vs. 71.0 ± 12.8 μm, *p* < 0.001). No differences were observed in the macular ganglion cell-inner plexiform layer (GCIPL) thickness between the two groups (72.2 ± 6.5 μm vs. 72.9 ± 5.4 μm, *p* = 0.558) [28]. In agreement with this, Moghadas Sharif et al. found no differences in the central ganglion cell layer (GCL) thickness between tilted and non-tilted high myopic subjects with an AXL > 26 mm (15 μm vs. 15 μm, *p* = 0.84) using Spectralis-OCT (Spectralis; Heidelberg Engineering, Heidelberg, Germany) [29]. Therefore, both groups agreed on the superior glaucoma diagnostic capability of the GCL thickness compared to the RNFL thickness and ONH parameters in the myopic tilted disc patients.

## 4. Optic Nerve Head Torsion

This represents the rotation of the ONH around the sagittal axis, commonly measured as the angle between the vertical meridian and the longest diameter of the optic disc on fundus photography [30]. More recently, the OCT-based torsion angle was defined as the measurement between the vertical meridian of the line connecting the center of the BMO opening and fovea and the longest diameter of the BMO-delineated ONH margin defined by SD-OCT [31]. Cheng et al. described OCT-based torsion angles of 9.1 ± 7.4°, 10.7 ± 9.1°, and 11.5 ± 11.1° in healthy subjects with AXL ≤ 25 mm, from 25 to 26 mm, and ≥26 mm, respectively [32]. Using the Spectralis OCT, Rezapour et al. found a torsion angle of 34.4° (range 29.9 to 39.0) vs. 33.7° (range 27.6 to 39.9) in mild myopia (mean AXL of 24.8 mm, range 24.7 to 25.0) vs. high myopia (mean AXL of 26.8 mm, range 26.6 to 27.0). In addition, they obtained no association between the photograph-based and OCT-based assessment of the torsion angle in high-axial myopic eyes (*p* ≥ 0.33 with R^2^ = 0.03 from 0.0 to 0.21) [33]. It should be highlighted that these different torsion angle values are due to the fact that the OCT-based torsion angle is a BMO-based assessment while the photographed-based torsion angle is measured relative to the clinical disc margin, which does not always correspond with the BMO, especially in myopic eyes.

## 5. Optic Disc Pits

OCT technology development has helped identify the optic disc pit in normal eyes [34]. In 2007, Shimada et al. first described the optic disc pit in myopic eyes using an OCT ophthalmoscope (C7, NIDEK, Gamagori, Aichi, Japan) [35]. Later, Ohno-Matsui et al. identified the pits using en-face images through vertical and horizontal SS-OCT scans in 16.2% (32/198) of highly myopic eyes (mean AXL: 30 ± 2 mm). Using en-face images, the pits can be observed, such as a triangular hyporeflective shape with the apex heading into the interior of the ONH. In horizontal and vertical OCT images, the optic disc pits were associated with discontinuities of the LC and discontinuous overlying RNFL. The pits were localized in the outer border of the ONH in 34% of cases and the scleral crescent in 64% of cases, being more frequently observed in the temporal conus. Remarkably, in this study, the pits were visible by fundus retinography in only two cases [36].

## 6. Peripapillary Intrachoroidal Cavitation

**Figure 3:**
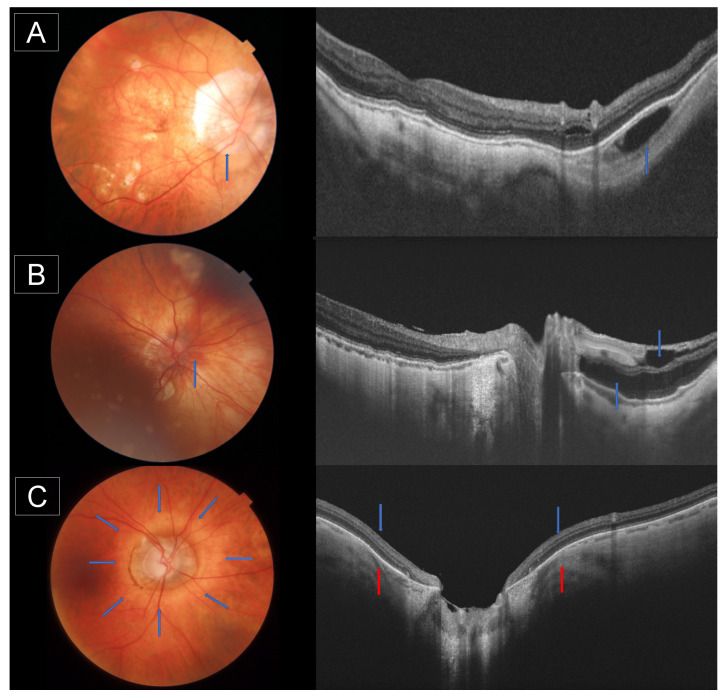
Fundus photography and optical coherence tomography (OCT) of the peripapillary region of a myopic subject. (**A**): Blue arrow represents peripapillary intrachoroidal cavitation observed in the photography as a yellowish peripapillary lesion and in the OCT image as a hyporeflective triangular thickening of the choroid with the base at the optic disc border, (**B**): Blue arrow represents peripapillary retinoschisis observed in the OCT image as cystoid hyporeflective spaces in the peripapillary region around retinal vessels, (**C**): Fundus photography and OCT image of peripapillary staphyloma, blue arrows showing the border of the staphyloma with arched posterior sclera and red arrows showing the thinned choroid at the edge of the staphyloma.

Based on OCT manifestations, peripapillary intrachoroidal cavitation (PICC) was first described by Freund et al. as a peripapillary detachment in pathologic myopia [37]. Later, Spaide et al. described the PICC as a suprachoroidal separation using SS-OCT [38]. Recently, Ehongo et al., also based on SS-OCT findings, suggested that the tensile forces of the optic nerve sheaths during adduction cause the collapse of the scleral flange onto the subarachnoid space, leading to PICC [39]. In OCT images, PICC is indeed defined as a hyporeflective triangular thickening of the choroid with the base at the optic disc border (Figure 3A), excluding the peripapillary large choroidal vessels. In their study, which evaluated 884 eyes of highly myopic subjects using SD-OCT (Model Ivue100; Optovue, Fremont, CA, USA), Liu et al. found PICC in 3.6% (32 eyes), frequently affecting the inferior disc (85.7%), followed by multiple locations (9.4%) and superior disc borders (3.1%) [40]. Similarly, Shimada et al. reported PICC in 4.9% of 324 subjects with pathologic myopia [35]. The Beijing Eye Study, however, reported a higher rate (16.9%) [41]. Moreover, Venkatesh et al. found PICC in 55.8% of highly myopic eyes with the presence of a myopic conus and/or the presence of intrascleral vessels near the cavitation [42]. You et al. measured PICC using OCT and showed that the mean width was 4.2 ± 2.3 h of the disc circumference, the mean length being 1363 ± 384 μm [41]. Older age, more myopic spherical equivalent, longer AXL, severe myopic maculopathy, and the presence of posterior staphyloma were associated with the presence of PICC [40,42].

## 7. Peripapillary Retinoschisis and Holes

Peripapillary retinoschisis (PPRS) is observed in OCT images as cystoid hyporeflective spaces in the peripapillary region around the retinal vessels (Figure 3B) [43]. Shimada et al. demonstrated retinal cysts in 49.5% of 287 highly myopic eyes (AXL > 26.5 mm), only 24.4% of which had been discovered by fundoscopy [44]. In agreement with this, Li et al. found PPRS in 109 eyes (71.7%) with an AXL > 26.5 mm [45]. A higher incidence of PPRS was associated with older age, longer AXL, and the presence of posterior staphyloma [44,45,46]. Interestingly, Li et al. revealed that the presence of PPRS was negatively associated with the MCT (β = −18.30, *p* < 0.05) in a recent study that included 645 myopic eyes [47].

The incidence of paravascular lamellar holes in highly myopic eyes ranges between 14% and 31.4%, according to different OCT scan protocols [44,48,49]. Vela et al. described that the holes are mainly distributed in the inferior temporal arcade in 39.9% and are related to staphyloma type V and IX [49]. The pathogenesis of the holes is unknown, although Shimada et al. suggested that the formation of the holes is probably due to the vitreous traction or spontaneous rupture of a cyst [44]. Interestingly, this OCT finding is proposed as an important causative factor for macular retinoschisis as it was noted in 43.1% to 80% of eyes with macular retinoschisis [44].

## 8. Peripapillary Staphyloma

Peripapillary staphyloma is traditionally evaluated by fundus photography or magnetic resonance imaging (MRI) scans [50]. In 2016, Shinohara et al. described the staphyloma on SS-OCT images in highly myopic eyes (mean AXL 31.1 ± 1 mm), which is characterized by an arched posterior sclera with less curvature in the adjacent regions (Figure 3C). The choroid at the edge of the staphyloma is thinned and is associated with PICC in 52.5% of the cases [51]. Peripapillary staphyloma can also be observed in young myopic subjects. Using ultra-widefield SS-OCT, Tanaka et al. observed the presence of staphyloma in 12.7% of the eyes of subjects younger than 20 years, with a mean AXL of 27.9 mm. Interestingly, they found that the subfoveal choroid and nasal choroid to the ONH were thinner in eyes with a staphyloma than those without [52]. In a recent study of 729 eyes with a mean AXL of 30 ± 2 mm, Shinohara et al. detected posterior staphyloma using ultrawide-field SS-OCT in 482 eyes (66.1%), and it was more frequently detected in eyes with macular retinoschisis (86.0% vs. 61.6%; *p* < 0.001) compared to eyes without macular retinoschisis [53]. It is of clinical relevance that the staphyloma is associated with a greater risk of glaucoma and VF loss [51].

## 9. Lamina Cribosa in Myopia

A large LC curvature, LC with a reduced thickness, and the presence of focal LC defects have been shown to correlate with highly myopic subjects [54,55,56] Using SS-OCT, Miki et al. observed LC defects in eight eyes with high myopia but without glaucoma (22.9%) vs. 28 eyes (41.8%) in high myopia with glaucoma (*p* = 0.0009). In this study, 79.5% of patients with LC defects had corresponding damage in the VF. Interestingly, other factors, such as visual acuity, intraocular pressure, disc ovality, or a PPA area, did not differ significantly between eyes with and without LC defects [54]. Sawada et al. used EDI SD-OCT B-scans to classify the defects into LC defects (with a diameter > 100 μm) and large pores with a diameter of 60 to 100 μm. They demonstrated that highly myopic eyes with glaucoma had more LC defects and larger pores than myopic eyes without glaucoma (3.8 vs. 0.8 and 1.9 vs. 1.6, respectively), both being more commonly located on the temporal side of the ONH. Interestingly, in this study, the number of temporal LC defects was associated with paracentral VF scotoma, whereas the number of inferior and superior LC defects was associated with the presence of superior and inferior VF defects [55].

Similarly, Han et al. used EDI SD-OCT to classify LC defects in myopic eyes into LC holes that were defined as a localized discontinuity of the LC, or LC disinsertion type defects defined as a posteriorly displaced laminar insertion with a downward slope at the far periphery of the LC toward the neural canal wall. Moreover, they noted more LC defects in myopic eyes with glaucoma than without glaucoma (65.7% vs. 27.8%, *p* < 0.001). These disinsertion-type LC defects were associated with the presence of glaucoma, AXL, and disc tilt angle, and they were found at the γ- PPA zone (R = 0.71, *p* < 0.001), while the location of hole-type LC defects did not correlate with the location of the γ-zone PPA (R = 0.07, *p* = 0.73) [56]. Table 2 summarizes the qualitative and quantitative measurable ONH characteristics in myopic subjects.

## 10. RNFL Measurement in Myopic Eyes: Anatomy

As the AXL increases, the retina is shifted temporally, resulting in the thickening of the RNFL in the temporal quadrant and its thinning in the other quadrants, especially the nasal [57]. In the superior-temporal region, the RNFL trajectories are associated with the course of the retinal vessels, while in the inferior-temporal region, they are associated with the course of the retinal vessels and disc torsion. This implies that, in 7.5% of the myopes, the RNFL raphe rotated enough to produce a false nasal step in the VF and is, generally, in poor agreement with the ISNT rule (67% in high myopes vs. 8% in emmetropes) [8]. In a population-based study of 5387 participants, Wagner et al. described that the angle between the peaks of the peripapillary RNFL in the upper and lower hemispheres decreased by, −5.86° with a 1 mm increase in AXL [58].

## 11. Factors Affecting RNFL Measurement

The peripapillary RNFL thickness measurement is one the most useful parameters to distinguish abnormal eyes in multiple ocular diseases. Measuring the RNFL thickness in myopic eyes is challenging as many factors may affect the measurements. Segmentation errors may occur due to incorrect delineation of the retinal layers, reaching up to 46.3% of the OCT scans [59]. Such artifacts are especially likely to occur in high myopia due to the anatomical features of the myopic ONH, such as extensive PPA, that makes RNFL recognition difficult, or due to the presence of intraretinal cyst-like anomalies that generate a false thickening of the RNFL [60]. Suwan et al. observed that manual correction was necessary for 32% vs. 56% of RNFL OCT scans of myopic eyes and myopic eyes with glaucoma, respectively (*p* < 0.001). Additionally, they showed that the glaucoma diagnostic capability of the global RNFL increased significantly after this correction, increasing the areas under the curve (AORUC) from 0.827 to 0.886 (*p* = 0.017) [61].

In addition, most commercial OCT devices do not include a database of highly myopic patients, which leads to false out-of-normal limit results when compared with healthy subject databases. In a study conducted on 193 healthy myopic eyes, 52 (26.9%) of the subjects showed at least one false positive red sign (*p* > 0.001) on the RNFL thickness map when comparing the measurements with the non-myopic data. This percentage increased to 62.8% in the case of high myopia (AXL > 26 mm) [60]. Seol et al., in a comparative validity study, implemented a myopic database to improve the Cirrus SD-OCT (Cirrus SD-OCT Carl Zeiss Meditec Inc) diagnostic ability in myopic glaucoma. The myopic normative database showed a higher specificity than the built-in normative database according to the quadrant RNFL thickness, clock-hour RNFL thickness, and GCIPL thickness (71% vs. 91%, 53% vs. 81%, and 66% vs. 91%, respectively; all *p* < 0.001) [62].

Similarly, the mean RNFL thickness may be affected by the magnification due to an increased AXL. This is because OCT devices have been configured to measure the RNFL thickness at a fixed angular distance, centered on the ONH, leading to a larger measuring circle when the AXL increases [63].

A large myopic optic disc may overestimate the RNFL thickness due to a shorter distance from the OCT scanning circle to the disc margin as the RNFL thickness becomes thinner with the increasing distance from the disc margin. Seo et al., using a Cirrus HD-OCT in 168 young myopic subjects, found that the average OCT RNFL thickness increased significantly with the optic disc area (5.35 μm/mm^2^, *p* < 0.001), while there was no significant correlation between the average GCIPL thickness and the ONH area [64].

A high optic disc tilt angle in myopic subjects may cause errors in the examination of the peripapillary area due to the difficulty in delaminating the center of the optic disc and the center of the opening of the BM, both being centers usually used to perform the RNFL thickness calculation. Additionally, eyes with optic tilt tend to have a thicker temporal RNFL thickness due to retina convergence towards the macular region [65]. Moghadas Sharif et al. found a significantly thicker temporal RNFL thickness in highly myopic subjects (AXL > 26 mm) with tilted ONH vs. no tilted ONH (29 μm vs. 25 μm *p* = 0.004) [29], while no significant differences were demonstrated in the average RNFL thickness between the tilted and non-tilted ONH [29,66] Finally, the presence of peripapillary detachment in pathologic myopia may lead to a misidentification of the outer profile of the RNFL. Kamal Salah et al. observed a thicker global RNFL thickness in myopic subjects with and without peripapillary detachment (88.2 ± 25 μm vs. 72.7 ± 16 μm, *p* = 0.001) [67].

## 12. RNFL Thickness Measurement by OCT

The mean RNFL thickness is usually reduced in highly myopic patients compared to emmetropic patients (89.8 ± 9 μm vs. 110.9 ± 10 μm, respectively, *p* > 0.001) [68]. Therefore, the RNFL thickness is negatively correlated with the AXL. Singh et al. and Kang et al. found that the mean RNFL thickness decreased by 3.74 μm and 2.2 μm, respectively, for each mm increase in the AXL [69,70] Additionally, this thinning of the RNFL in highly myopic eyes is not uniformly distributed, being greater in the lower quadrant (134.8 ± 17 μm vs. 109.9 ± 15 μm, *p* > 0.001) in emmetropes vs. high myopes, respectively. It has also been observed that myopic eyes have a significantly greater rate of decline in RNFL during follow-up than controls. In subjects between 30 and 39 years old, Lee et al. observed that the RNFL loss was 0.95 μm/year vs. 0.57 μm/year in myopic vs. healthy subjects; 1.69 μm/year vs. 0.48 μm/year in myopic vs. healthy subjects aged 40 to 49, and the loss rate was 1.70 μm/year vs. 0.63 μm/year in myopic vs. healthy subjects in the group from 50 to 59 years [71]. Similarly, myopic subjects with glaucoma have a higher rate of RNFL loss than non-myopic glaucoma subjects. Biswas et al. found that, during follow-up (>60 months), myopic eyes with AXL ≥ 26.0 and ≥26.5 mm had an average rate loss in RNFL thickness of 0.15 and 0.16 μm/year, faster than eyes with AXL < 26 and 26.5 mm, respectively [72].

Taking into account all of the aforementioned factors, the peripapillary RNFL thickness should be used with caution to distinguish healthy from glaucoma highly myopic subjects. Seo et al. showed that the best RNFL sectors for diagnosing glaucoma in patients with high myopia were the temporal-inferior sector and the inferior quadrant (AUROC were 0.974 and 0.951, respectively) [64]. Similarly, Rolle et al., using Fourier-Domain-OCT (FD-OCT RTVue-100; Optovue, Fremont, CA, USA), found that the average RNFL yielded the best diagnostic ability for the diagnosis of glaucoma in patients with AXL > 25 mm, followed by the superior and inferior RNFL, reaching AUROC of 0.883, 0.858, and 0.872, respectively [73]. As the macular parameters are not significantly affected by high myopia, recent studies have established that the macular ganglion cells’ layer thickness has higher diagnostic power and progression analysis than the peripapillary RNFL thickness in high myopia [74,75,76], the inferotemporal macular GCIPL thickness being the best parameter for myopic glaucoma discrimination [74].

## 13. Bruch’s Membrane Opening-Minimum Rim Width

**Figure 4:**
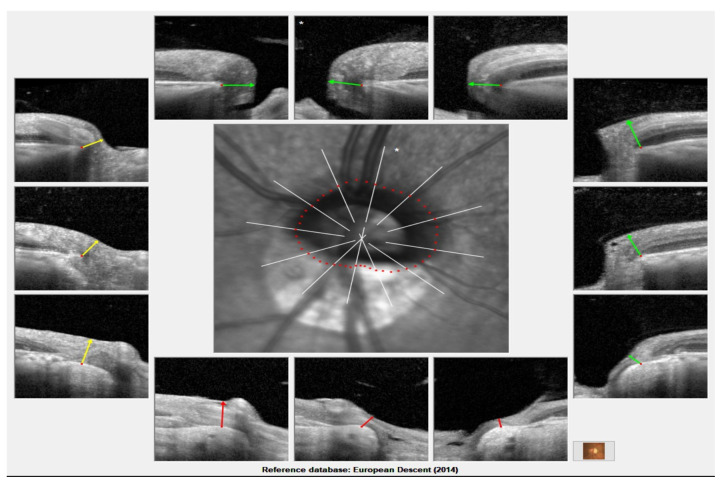
Bruch’s membrane opening-minimum rim width (BMO-MRW) measurement in a high myopic patient. Green, yellow, and red lines represent the least distance between Bruch’s membrane opening and the internal limiting membrane. Green, yellow, and red color of the lines represent the classification of BMO-MRW measurement within normal limits, borderline and out of normal limits, respectively.

The Bruch’s membrane opening-minimum rim width (BMO-MRW) represents the minimum distance between the BM opening and the internal limiting membrane (Figure 4). BMO-MRW measurements may have higher accuracy in detecting glaucoma than RNFL [77,78]. Sastre-Ibañez et al. reported no correlation between the AXL and BMO-MRW in moderate myopic subjects. In this study, which used Spectralis SD-OCT and Glaucoma Premium Module Edition (GPME) software version 6.0c, they showed that the number of sectors classified as being outside of the normal limits was significantly lower compared to the RNFL analysis 0.2 ± 0.6 vs. 0.7 ± 1.1, respectively (*p* = 0.023) [79]. Wang et al. found that the BMO-MRW classified a significantly lower percentage of eyes as being outside the normal limits in at least one quadrant than the RNFL thickness (4% vs. 34.67%; *p* < 0.01) [80]. Similarly, Uzair et al. noted that the specificity was better with the OCT BMO-MRW (85.7%) than with the RNFL (66.7%). Moreover, they observed a higher agreement between the glaucoma expert classification and BMO-MRW based classification (κ = 0.800, *p* < 0.001), rather than the RNFL-based classification (κ = 0.480, *p* < 0.001), to identify subjects outside of the normal limits [81]. This indicated that BMO-MRW measurement reduced the false-positive rate caused by myopia. Table 3 summarizes the characteristics of the RNFL measurement in highly myopic subjects.

## 14. Conclusions

The diagnosis of glaucoma and its severity by ONH imaging in highly myopic subjects is still a key challenge in clinical practice. In recent years, OCT has become the mainstay for ONH study, offering high-resolution images of the papillary and peripapillary region in highly myopic eyes. This imaging technique allows clinicians to describe the qualitative typical characteristics of the myopic ONH and quantify them with measurable parameters. A larger β-PPA area is associated with older age, a longer AXL, and thinner choroidal thickness. ONH tilt is more frequent in myopic eyes and is usually greater in myopic eyes with glaucoma, with a negative correlation with VF defects. Peripapillary intrachoroidal cavitation is associated with severe myopic maculopathy and the presence of posterior staphyloma. Myopic eyes with glaucoma have larger and more LC defects than eyes without glaucoma.

A correction of segmentations errors and the use of a myopic database should be considered to increase the ability of RNFL measurement to distinguish eyes outside of the normal range. Both the macular GCL and BMO-MRW may reduce the false-positive results caused by myopia and are promising parameters for the diagnosis of glaucoma. The OCT results were obtained from transversal or retrospective studies with no comparative study samples. Therefore, future prospective studies with large samples are needed to confirm the aforementioned findings. It will be interesting to have a standard scanning protocol in the future to explore ONH in myopic eyes.

## Figures and Tables

**Table 1:** ONH characteristics in myopic subjects.

*α-PPA*	Irregular RPE with the presence of BM
*β-PPA*	Absence of the RPE and the presence of BM
*γ-PPA*	Absence of both the RPE and BM with the presence of only the peripapillary RNFL
*δ-PPA*	Absence of microvessels larger than 50 microns usually within the *γ-PPA* zone
ONH tilt angle	Angle between two lines: the first line connecting the inner edges of BM on each side of the ONH on the cross-sectional OCT image and a second line connecting the two points of the clinical disc margin along the OCT cross-sectional scan
ONH torsion angle	Angle between the vertical meridian of the line connecting the center of the BMO and fovea and the longest diameter of the BMO-delineated ONH margin defined by OCT
ONH pits	Triangular hyporeflective shape with the apex heading into the interior of the ONH observed in enface images
PICC	Hyporeflective triangular thickening of the choroid with the base at the optic disc border excluding peripapillary large choroidal vessels
PPRS	Cystoid hyporeflective spaces in the peripapillary region around retinal vessels
Peripapillary staphyloma	Arched posterior sclera with less curvature in adjacent regions, the choroid at the edge of the staphyloma is thinned

ONH: optic nerve head; PPA: peripapillary atrophy; RPE: retinal pigment epithelium; BM: Bruch’s membrane; RNFL: retinal nerve fibre layer; OCT: optical coherence tomography; BMO: Bruch’s membrane opening; PICC: peripapillary intrachoroidal cavitation; PPRS: peripapillary retinoschisis.

**Table 2:** Measurable ONH characteristics in myopic subjects.

PPA area	0.35 mm^2^ in AXL < 24 mm [18] 0.65 mm^2^ in AXL 24 to 27 mm [18] 0.78 mm^2^ in AXL > 27 mm [18] 0.1 mm^2^ increase in PPA area for every 14.93-μm decrease in MCT [18] 0.1 mm^2^ increase in PPA area for every 9.54-μm decrease in peripapillary CT [18] Week correlation with RNFL (R = 0.417, *p* < 0.001) [19]
ONH Tilt	Tilt angle 3.5° (1.2–11.2) [22] Temporal tilt angle 3.60° (1.61–6.40°) [23] Vertical tilt angle 0.00° (−1.03° to 1.62°) [23] Tilt in myopic glaucoma vs. myopic 9.3 ± 6.3° vs. 6.2 ± 4.1° (*p* < 0.05) [24] Tilt angle in myopic glaucoma during 3 years of follow-up 8.3 ± 3.8° vs. 7.0 ± 3.4 [25] Tilt angle correlation with VF (r = −0.356, *p* < 0.001) [22] Lower deviation of the VF in the superotemporal quadrant (−4.54 ± 3.16 dB) in myopic with tilted ONH [26] Myopic tilted vs. non-tilted myopic [28] ○ Disc area 1.91 ± 0.81 mm^2^ vs. 1.63 ± 0.30 mm^2^ (*p* < 0.05) ○ Cup volume 0.53 ± 0.28 mm^2^ vs. 0.33 ± 0.19 mm^2^, (*p* < 0.05) ○ CDR 0.76 ± 0.09 vs. 0.69 ± 0.11, (*p* < 0.05) ○ Temporal RNFL 62.1 ± 9.0 μm vs. 71.0 ± 12.8 μm, (*p* < 0.001) ○ GCIPL 72.2 ± 6.5 μm vs. 72.9 ± 5.4 μm, (*p* = 0.558)
ONH torsion	Torsion angle 9.1 ± 7.4° AXL ≤ 25 mm [32] Torsion angle 10.7 ± 9.1° AXL from 25 to 26 mm [32] Torsion angle 11.5 ± 11.1° AXL ≥ 26 mm [32] Torsion angle 34.4° (range 29.9 to 39.0) vs. 33.7° (range 27.6 to 39.9) in mild 24.8 mm (range 24.7 to 25.0) vs. high 26. 8 mm (range 26.6 to 27.0) myopic eyes [33]
ONH pits	16.2% of high myopic eyes (AXL 30 ± 2 mm) [36] ○ 34% localized in the outer border of the ONH ○ 64% localized in the scleral crescent
PICC	PICC in 3.6% of myopic eyes [40] ○ Inferior disc border (85.7%) ○ Multiple locations (9.4%) ○ Superior disc borders (3.1%). PICC in 4.9% of pathologic myopia eyes [35] PICC in 16.9% of myopic eyes [41] PICC mean width = 4.2 ± 2.3 h of the disc circumference [41] PICC mean length = 1363 ± 384 μm [41]
PPRS	PPRS 49.5% of high myopic eyes (AXL > 26.5) [44] PPRS (71.7%) high myopic eyes with AXL > 26.5 mm [45] PPRS presence was negatively associated with MCT (β = −18.30, *p* < 0.05) [47]
PP hols	The incidence ranges between 14% and 31.4% [44,47,48,49] 39.9% distributed in the inferior temporal arcade [49] Related to staphyloma V and IX [49] Found in 43.1% of eyes with macular retinoschisis [49] Found in 80.0% of eyes with macular retinoschisis [44].
PP staphyloma	12.7% of eyes of subjects younger than 20 years with mean AXL of 27.9 mm [52] 66.1% of eyes with mean AXL of 30 ± 2 mm [53] 86.0% vs. 61.6% (*p* < 0.001) in eyes with macular retinoschisis vs. without macular retinoschisis [53] Associated with PICC in 52.5% of the cases [51]
LC	Present in 22.9% vs. 41.8% (*p* < 0.05) in high myopic vs. high myopic with glaucoma [54] Present in 27.8% vs. 65.7% (*p* < 0.001) in high myopic vs. high myopic with glaucoma [56] 79.5% of patients with LC defects had corresponding damage in VF [54] The number of LC defects with a diameter > 100 μm: 3.8 vs. 0.8 in myopic eyes with vs. without glaucoma [55] The number of large pores with a diameter of 60 to 100 μm: 1.9 vs. 1.6 in myopic eyes with vs. without glaucoma [55] Disinsertion-type LC defects were associated with glaucoma, AXL, and disc tilt angle (R = 0.71, *p* < 0.001) [56]

ONH: optic nerve head; PPA: peripapillary atrophy; AXL: Axial length; MCT: macular choroidal thickness; CT: choroidal thickness; RNFL: retinal nerve fiber layer; VF: visual field; CDR: cup to disc ratio; GCIPL: macular ganglion cell-inner plexiform layer; PP: peripapillary; PPIC: peripapillary intrachoroidal cavitation; PPRS: peripapillary retinoschisis; LC: lamina cribrosa.

**Table 3:** Characteristics of RNFL measurement in highly myopic subjects.

Segmentations errors up to 46.3% of the OCT scans [59]. The manual correction was necessary for myopic eyes and myopic eyes with glaucoma in 32% vs. 56% (*p* < 0.001) of the RNFL scan [61]. 26.9% of the subjects showed at least one false positive red sign on the RNFL thickness map based on non-myopic data [82]. 62.8% of subjects with AXL > 26 mm showed at least one false positive red sign on the RNFL thickness map based on non-myopic data [82]. RNFL specificity of glaucoma diagnosis: 71% vs. 91% (*p* < 0.001) myopic normative database vs. built-in normative database [62]. RNFL thickness: 88.2 ± 25 μm vs. 72.7 ± 16 μm, (*p* = 0.001) in myopic subjects with and without peripapillary detachment [67]. The mean RNFL thickness: 110.9 ± 10 μm vs. 89.8 ± 9 μm in highly myopic patients compared to emmetropic patients (*p* < 0.001) [69]. RNFL thickness decreased by 3.74 μm for each mm of increase in AXL [69]. RNFL thickness decreased by 2.2 μm for each mm of increase in AXL [70]. Progressive thinning of RNFL in myopic vs. non-myopic subjects [71] ○ Aged 30 to 39 years old: 0.95 μm/year vs. 0.57 μm/year ○ Aged 40 to 49 years old: 1.69 μm/year vs. 0.48 μm/year ○ Aged 50 to 59 years old: 1.70 μm/year vs. 0.63 μm/year Best diagnostic ability of glaucoma in patients with high myopia [64] ○ Temporal-inferior sector: AUROC of 0.974 ○ Inferior quadrant: AUROC of 0.951 [64] Best diagnostic ability of glaucoma in patients with AXL > 25 mm [73] ○ Average RNFL: AUROC of 0.883 ○ Superior RNFL: AUROC of 0.858 ○ Inferior RNFL: AUROC 0.872 The number of sectors classified as outside of normal limits in BMO-MRW classification vs. RNFL classification 0.2 ± 0.6 vs. 0.7 ± 1.1 (*p* = 0.023) [79]. Percentage of eyes as outside normal limits in BMO-MRW classification vs. RNFL classification: 4% vs. 34.67% (*p* < 0.01) [80]. Specificity for glaucoma diagnosis in BMO-MRW classification vs. RNFL classification: 85.7% vs. 66.7% [81]. No correlation between AXL and BMO-MRW in moderate myopic subjects [79].

RNFL: retinal nerve fibre layer; OCT: ocular coherence tomography; AXL; axial length; AUROC: area under the receiver operating characteristic; BMO-MRW: Bruch’s membrane opening-minimum rim width.

## Data Availability

Not applicable.
